# The Synchronized Efforts to Decipher the Molecular Basis for Soybean Maturity Loci *E1*, *E2*, and *E3* That Regulate Flowering and Maturity

**DOI:** 10.3389/fpls.2021.632754

**Published:** 2021-04-28

**Authors:** Zhengjun Xia, Hong Zhai, Hongyan Wu, Kun Xu, Satoshi Watanabe, Kyuya Harada

**Affiliations:** ^1^Key Laboratory of Soybean Molecular Design Breeding, Northeast Institute of Geography and Agroecology, The Innovative Academy of Seed Design, Chinese Academy of Sciences, Harbin, China; ^2^Faculty of Agriculture, Saga University, Saga, Japan; ^3^Department of Biotechnology, Graduate School of Engineering, Osaka University, Suita, Japan

**Keywords:** soybean, flowering time, maturity, photoperiodic response, positional cloning, *E1*

## Abstract

The general concept of photoperiodism, i.e., the photoperiodic induction of flowering, was established by Garner and Allard (1920). The genetic factor controlling flowering time, maturity, or photoperiodic responses was observed in soybean soon after the discovery of the photoperiodism. *E1*, *E2*, and *E3* were named in 1971 and, thereafter, genetically characterized. At the centennial celebration of the discovery of photoperiodism in soybean, we recount our endeavors to successfully decipher the molecular bases for the major maturity loci *E1*, *E2*, and *E3* in soybean. Through systematic efforts, we successfully cloned the *E3* gene in 2009, the *E2* gene in 2011, and the *E1* gene in 2012. Recently, successful identification of several circadian-related genes such as *PRR3a*, *LUX*, and *J* has enriched the known major *E1-FTs* pathway. Further research progresses on the identification of new flowering and maturity-related genes as well as coordinated regulation between flowering genes will enable us to understand profoundly flowering gene network and determinants of latitudinal adaptation in soybean.

## Introduction

In plants, various external cues, e.g., day length and temperature, can trigger endogenous physiological changes and lead to flowering, the critical change from vegetative growth stage to maturity stage. Garner and Allard (1920) discovered “photoperiodism” describing that day length can influence flowering time in many plant species (Garner and Allard, 1920). Along with tobacco and other plants, soybean was used as a model plant that greatly contributed to the advances of photoperiodism (Garner and Allard, 1920; Owen, 1927; Heinze et al., 1942). As the most important external cues, light is received by photoreceptors, e.g., phytochromes, cryptochromes, and phototropins. The functions of the phytochromes, the red light and far-red light absorbing photoreceptors, in initiation of flowering were extensively studied (Takimoto and Hamner, 1965). As early as in 1934, the leaf was found to sense day length (Knott, 1934). Florigen is proposed for the signal that is transmitted from leaves to the shoot apical meristem (SAM) where the flowering is initiated (Chailakhyan, 1936). Recent molecular advances have identified that FT protein, a rather small protein with a certain similarity to RAF kinase inhibitors (Kardailsky et al., 1999; Kobayashi et al., 1999), functions as Florigen, which is produced in leaves and transmitted to the SAM (Corbesier et al., 2007; Jaeger and Wigge, 2007; Tamaki et al., 2007; Notoguchi et al., 2008). The molecular mechanism of flowering has been well understood using model plants, *Arabidopsis thaliana* and rice (*Oryza sativa*). Several regulatory network pathways controlling flowering have been deciphered (Amasino, 2010; Fornara et al., 2010). In *Arabidopsis*, *CONSTANS* (*CO*), *GIGANTEA* (*GI*), and *FLOWERING LOCUS T* (*FT*) have been proven to be central components for initiation of flowering in long-day conditions (Koornneef et al., 1991; Kardailsky et al., 1999; Fornara et al., 2010).

In soybean, nine maturity loci, known as E-series (*E1* to *E8*) and *J* conditioning flowering, have been identified and characterized genetically (Bernard, 1971; Buzzel, 1971; Buzzel and Voldeng, 1980; McBlain and Bernard, 1987; Ray et al., 1995; Bonato and Vello, 1999; Cober and Voldeng, 2001a; Cober et al., 2010). Recently, *E9*, *E10*, and *E11* of *E* series were nominated (Kong et al., 2014; Zhai et al., 2014a; Zhao et al., 2016; Samanfar et al., 2017; Wang et al., 2019).

The *E1*, *E3*, *E4*, and *E7* loci were proven to be photoperiod sensitive to different light quality conditions (Buzzel, 1971; Buzzel and Voldeng, 1980; McBlain et al., 1987; Cober et al., 1996a, b; Abe et al., 2003). Flowering delay under long-day for the alleles of *E1*, *E4*, and *E7* was conditioned by the light quality with lower red to far-red (R:FR) quantum ratios (Cober et al., 1996a; Cober and Voldeng, 2001b). However, the *E3* locus is less sensitive to light quality, which was revealed by similar flowering delays under long-day conditions with various light qualities (Cober et al., 1996a). The recessive *E3* allele conditions long-day insensitivity under fluorescent light with a high R:FR ratio (Buzzel, 1971), whereas *E4* needs the presence of *E3* to achieve long-day insensitivity in incandescent light with a low R:FR ratio (Buzzel, 1971; Buzzel and Voldeng, 1980). Particularly, the *E1* locus confers a largest effect on flowering time under various environmental conditions (Bernard, 1971; Abe et al., 2003; Stewart et al., 2003).

Characterization of isolines of *E* allelic combinations (Upadhyay et al., 1994a, b) revealed that each *E* locus exerts its influence on flowering time and maturity and also pleiotropic effects on some different developmental processes (Curtis et al., 2000), e.g., plant height and yield (Mansur et al., 1993; Chapman et al., 2003; Cober and Morrison, 2010).

**FIGURE 1:**
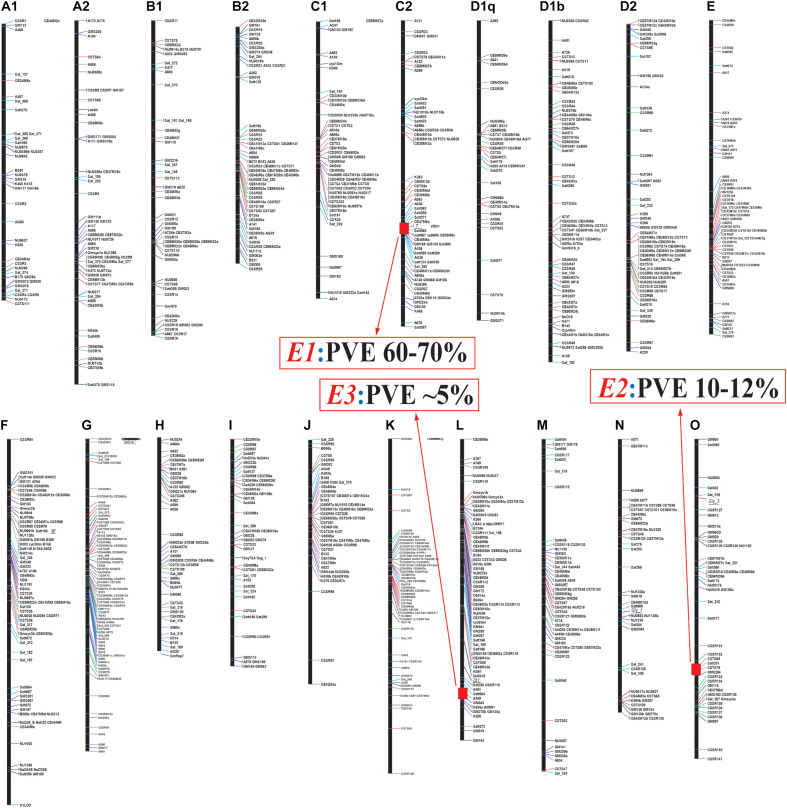
Linkage map construction using an F2 population derived from a cross between Misuzudaizu and Moshidou Gong 503 (adapted from Xia et al., 2007). Identified Quantitative trait loci (QTLs) of *E1, E2* and *E3* for flowering time were indicated by red segments. PVE, phenotypic variance explained by each QTL. Name of each linkage map is depicted on the top.

Until 2000, the molecular bases for *E* series had not been disclosed; therefore, Professor Kyuya Harada’s research team at Chiba University, Japan had started to develop recombinant inbred line (RIL) populations for linkage maps (Yamanaka et al., 2000), and quantitative trait locus (QTL) analyses (Yamanaka et al., 2001; Watanabe et al., 2004) toward deciphering the molecular basis for the *E1*, *E2*, and *E3* loci using the positional cloning strategy (Watanabe et al., 2009, 2011; Xia et al., 2012; Figure 1).

## The Method and Strategy of Residual Heterozygous Lines for Positional Cloning

### Mapping Population, Linkage Map, and QTL Mapping

Quantitative trait locus analysis (Tanksley, 1993) was employed to dissect the genetic factors for the quantitative trait flowering time into separate components by using RILs. The RILs were derived from a cross between Misuzudaizu, a Japanese variety, and Moshidou Gong 503, a weedy line from China.

A population of 156 RILs (F8:10) was used for QTL analysis of flowering. Three QTLs for flowering time, *FT1*, *FT2*, and *FT3* were, respectively identified at LG C2 (Chr. 6), LG O (Chr. 10), and LG L (Chr. 19), which were respectively corresponding to *E1*, *E2*, and *E3*, according to the map positions (Yamanaka et al., 2001; Watanabe et al., 2004; Xia et al., 2007; Figure 1). All the late-flowering alleles *E1*, *E2*, and *E3* were partially dominant over the early flowering alleles, *e1*, *e2*, and *e3*, respectively. The parent Misuzudaizu carried late-flowering allele at the *E1* and *E3* loci, whereas Moshidou Gong 503 had the late-flowering allele at the *E2* locus.

**FIGURE 2:**
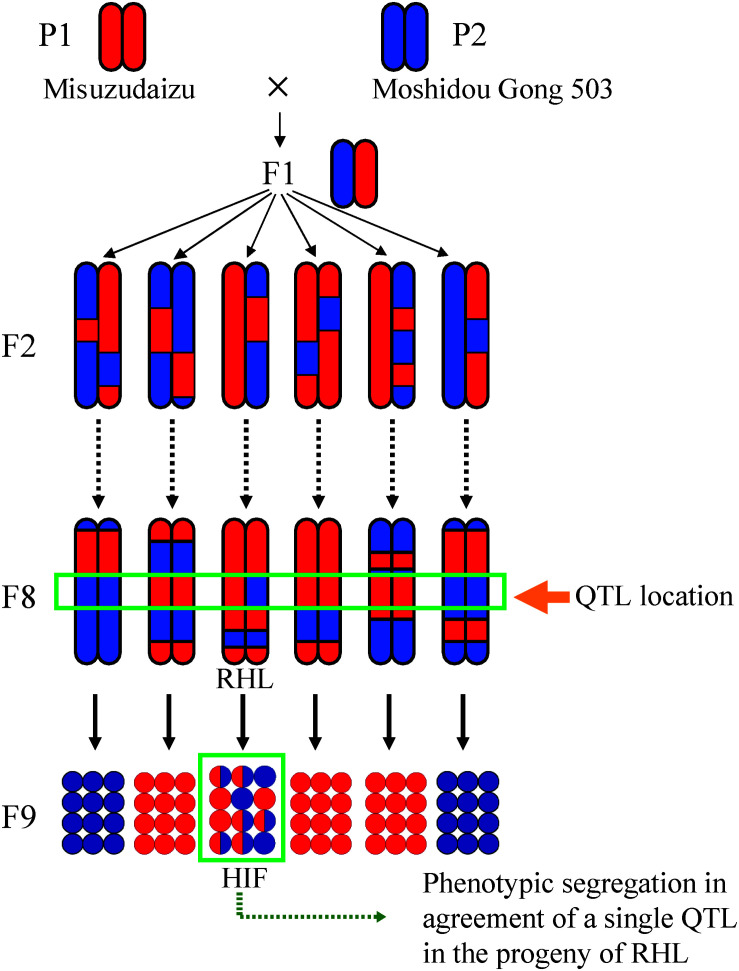
A schematic representation of residual heterozygous line (RHL) strategy for positional cloning. The RHL retains a heterozygous region including the target QTL region but carries homozygous regions across the genome especially for the other QTL regions detected. Meshed circles show heterozygous individuals. HIF refers to heterogeneous inbred family.

Although near-isogenic lines (NILs) that contain a QTL in a small, defined chromosomal region are beneficial for fine mapping of the QTL, however, developing NILs is rather difficult and time and labor intensive especially in soybean. Instead, residual heterozygous lines (RHLs) were employed in our fine mapping (Yamanaka et al., 2005; Figure 2). With a set of developed molecular markers, in an RIL population, we were able to identify a given RHL or a set of given RHLs harboring a heterozygous region encompassing a given target QTL but homozygous for the most other regions of the genome, especially for the other QTL regions for the same trait. Phenotypic segregation was generally observed in the progenies of the RHL, the pattern of which depends on the effects of the target QTL (Figure 2). Similarly, heterogeneous inbred family (HIF) defined by Tuinstra et al. (1997) was successfully used to identify the QTL associated with seed weight in sorghum (Tuinstra et al., 1997).

Genotypes of a given trait in recombinants identified in the progenies of RHL could be deduced from the segregation patterns in the next generation. Theoretically, the probability of successful identification of RHLs for a target QTL depends on the heterozygosity ratio and the size of the population studied (Figure 2). The formula of nC_*k*_p^*k*^ (1-p)^*n*–*k*^ can be used to calculate the possibility of the probability of successfully detecting k individuals with a heterozygous genotype at the target region, in which p is the ratio of heterozygosity of any population with given size of n. Taking an F7 generation of RILs as an example, the ratio of heterozygosity (p) is 0.0156; the probability of detecting at least one RHL in a population size of 200 is more than 0.95. In our practice, confirmed QTL analysis using the F6–F8 RIL population together with the RHL strategy is beneficial for unwinding genetic factors for an agronomic trait into each QTL (Figure 2).

### Marker Development

Since cloning of *E1*, *E2*, and *E3* genes started at the time before the soybean reference genome sequences of Williams 82 were available, amplified fragment length polymorphism (AFLP), simple sequence repeat (SSR), and sequence characterized amplified region (SCAR) markers were mainly used for developing new markers and genotyping a large population of the RHLs’ progenies (Xia et al., 2007; Watanabe et al., 2009).

In the given QTL region of RHL-derived population, recombinants were identified through DNA markers, whereas the genotypes of flowering time of recombinants were validated by progeny test. If the markers cosegregated with genotypes of flowering time, bacterial artificial chromosome (BAC) or transformation-competent bacterial artificial chromosome (TAC) clones compassing these markers were identified (Xia et al., 2005, 2009; Wadahama et al., 2008). Based on the fingerprinting profiles, BAC end sequencing, and relationships between BAC and markers, the BAC or TAC contig could be built. BAC clones covering the target region were selected for sequencing. The sequence data were assembled and annotated. Further functional confirmation of a candidate gene was carried out by association analysis, allelic variation, and gene disruption by induced mutation.

## The Route to Successful Identification of the *E3* Gene

**FIGURE 3:**
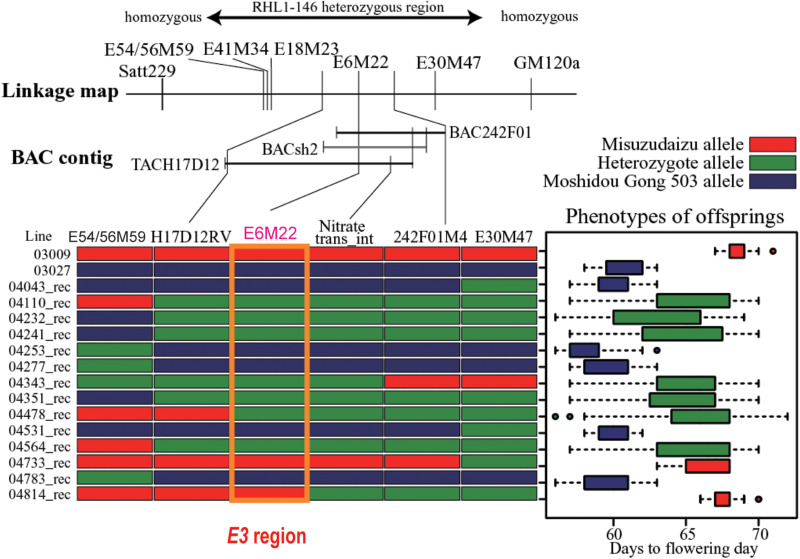
Fine mapping of the *E3* gene. The heterozygous region is shown on the top. The linkage map of markers and bacterial artificial chromosome (BAC) contig are displayed in the middle panel. Mapping using recombinants are shown in the bottom panel: left, recombinants detected; right, phenotypic segregation patterns in the progenies. Recombination is shown by red bars representing homozygous Misuzudaizu allele, blue bars representing homozygous Moshidou Gong 503, and green bars representing the heterozygote. The phenotypic segregation is shown in boxplot format. The delimited *E3* region is shown in the purple box (Adapted from Watanabe et al., 2009).

Totally, six DNA markers, including three AFLP-derived and three PCR-based markers developed from the BAC/TAC sequences, were employed for fine mapping of the *E3* locus. Through systematic fine mapping, it was strongly suggested the *E3* gene had been successfully delimited to the physical region covered by TACH17D12 (Figure 3).

Based on the sequence of GM_TMiH_H17D12, a total of 11 genes were predicted. Considering having a large effect on flowering time under FLD conditions, a candidate for the *E3* gene might be a photoreceptor (Cober et al., 1996a). The gene *GmPhyA3* encoding phytochrome A was considered to be a strong candidate for *E3*. This *E3* gene was referred to as *GmPhyA3*, following *GmPhyA1* and *GmPhyA2*, that had been assigned for other phytochrome A genes when the *E4* gene was cloned (Liu et al., 2008).

*GmPhyA3* from Misuzudaizu (*GmPhyA3*-Mi) encodes a 1130 amino acid protein. *GmPhyA3*-Mi carries normal conserved domains for phytochrome A type protein, including two Per/Arnt/Sim (PAS) domains, a histidine kinase domain, and a chromophore-attached domain. *GmPhyA3*-Mo from Moshidou Gong 503 carries a large insertion in the fourth intron and one functional single-nucleotide polymorphism (SNP) (glycine to arginine) in the third exon. Amazingly, this SNP was captured by AFLP technique as marker E6M22 (Figure 3). The insertion sequence is approximately 2.5 kb of the non-long-terminal-repeat (non-LTR) retrotransposon reverse transcriptase element, a portion of which is highly homogeneous to the *Ty1*/*copia* or *Ty1*/*gypsy* sequences in the *E4* allele (Liu et al., 2008).

**FIGURE 4:**
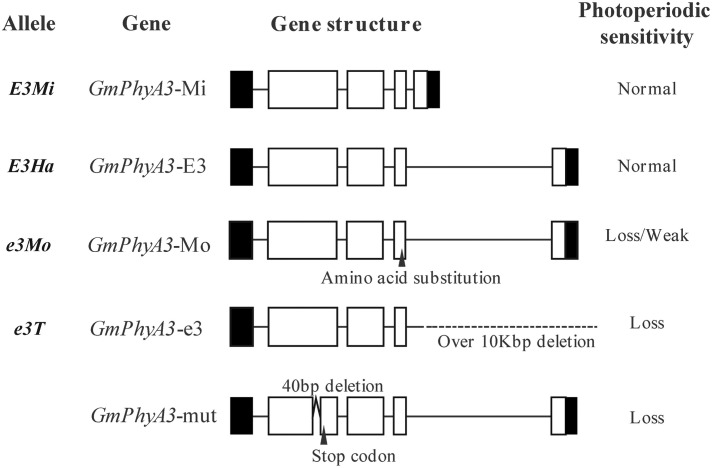
The allelic variation of the *E3* gene. Open boxes, shaded boxes, and horizontal lines, respectively indicate exons, untranslated regions (UTRs), and introns of protein structure. Variations such as deletion, insertion, and presence of the stop codon are indicated. On the right side are shown their photoperiod sensitivity.

*E3* gene sequences from Harosoy and Harosoy-*e3* were, respectively designated as *GmPhyA3-E3* and *GmPhyA3-e3* (Figure 4). In addition, a retrotransposon-like insertion sequence was also identified in *GmPhyA3-E3*, as well as in *GmPhyA3*-Mo (Figure 4). However, the amino acid sequences encoded by *GmPhyA3*-Mi and *GmPhyA3-E3* were identical.

Additionally, a large deletion of 13.33 kb occurred at the beginning of the third exon in *GmPhyA3-e3*. Furthermore, a mutant (*GmPhyA3*-mut), with a 40-bp deletion in *GmPhyA3* gene, was identified from the mutant libraries of Bay using targeting-induced local lesions in genomes (TILLING) (Figure 4; Watanabe et al., 2009).

Genetic analysis revealed that F2 population derived from a cross between Harosoy and 6-22-*ft3* showed a significant difference on flowering time in agreement with *E3* genetic effect, indicating the *E3* and *FT3* alleles are eventually identical.

In addition, large retrotransposon sequences inserted into *GmPhyA3-E3* and *GmPhyA3*-Mo might exert no noticeable effect on the phenotype, whereas the single AA substitution that occurred in the *GmPhyA*-Mo might have a weak effect on the *E3* allele (Figure 4; Watanabe et al., 2009).

Considering that a large effect under FLD had been reported for the *E3* allele (Cober et al., 1996b), the sensitivities of the three NILs (Harosoy and -*E3*, 6-22-*FT3* and -*ft3*, 1-146-*FT3* and -*ft3*) and the mutant line for the *GmPhyA3* gene to FLD conditions were evaluated. The result showed that the effect of the *E3* allele was promoted under FLD conditions in all the NILs, although different genetic backgrounds also can determine the basal line of flowering days. The *GmPhyA3*-mut mutant flowered 15 days earlier than the wild-type cultivar Bay under FLD mimic condition, in which sunlight was extended with a mercury-vapor lamp with high red/far-red (R/FR) ratio (Watanabe et al., 2009). Refer to the formal publication on the positional cloning of the *E3* gene (Watanabe et al., 2009) for the detailed cloning procedure.

Recently, Liu Y. et al. (2020) systematically illustrated the dynamic allelic variations in the *E3* gene based on pan-genome information of wild and cultivated soybean. In addition, the existence of a read-through type gene fusion between *E3* and its neighboring genes including *SoyZH13_19G210600* was demonstrated.

## The Route to Successful Identification of the *E2* Gene

**FIGURE 5:**
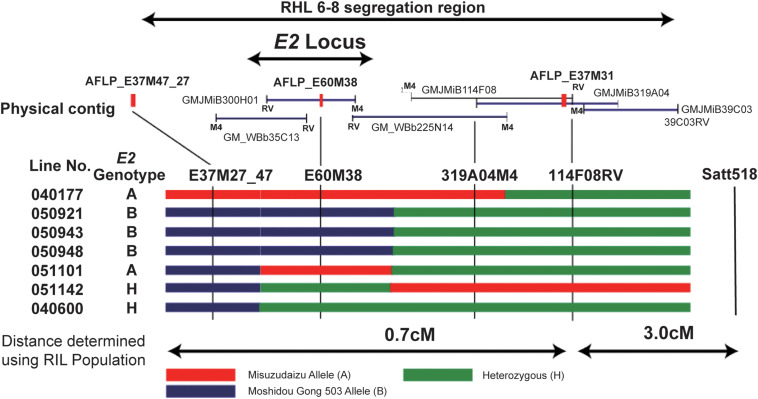
Fine mapping of the *E2* gene using residual heterozygous line (RHL) strategy. The segregation region of the line of RHL 6–8 are shown on the top. The physical contig are shown in the middle panel, in which the BAC or TAC clones and the developed markers are placed in the relative physical position. The M4 end or RV end is also indicated. Mapping using recombinants are shown in the bottom panel. Left: Recombinant line detected. Recombination is shown by black bars representing homozygous Misuzudaizu allele, white bars representing homozygous Moshidou Gong 503, and dotted bars representing the heterozygote. Genotypes of *E2* are judged based on the phenotypic segregation in the next generation. The delimited *E2* region is shown. Right: phenotypic segregation patterns in the following year in the progenies (Adapted from Watanabe et al., 2011).

The strategy that has been employed for cloning of the *E3* gene was used for cloning of the *E2* gene. The *FT2* locus corresponded to the maturity locus *E2* (Yamanaka et al., 2001). In the RIL population, the line RIL6-8 was identified to carry heterozygous region covering the *E2* locus; therefore, this line is hereafter referred to as RHL6-8 (Figure 5; Watanabe et al., 2011).

Three SCAR markers that had been successfully developed from these five polymorphic products were used to screen two independent BAC libraries, and a total of 10 BAC clones were acquired and a contig of approximately 430 kb was built (Watanabe et al., 2011). Three molecular markers, one AFLP-derived and two BAC-sequence-derived markers, were employed for the fine mapping to delimit the *E2* locus (Watanabe et al., 2011). The *E2* locus could explain 87.9% of the total variance in flowering time, indicating that a single QTL or gene controls this trait observed in this population. The marker 2 (E60M38) cosegregated with *E2* judging from the flowering time, indicating that this marker was physically close to *E2* (Watanabe et al., 2011). Judging from the phenotypes and genotyping data of recombinants as well as the positions where recombination events occurred, the *E2* locus could be delimited into the single BAC clone, MiB300H01 (Watanabe et al., 2011; Figure 5). The whole sequence of the BAC clone, MiB300H01, was determined using shotgun sequencing. Among the nine genes annotated for the 94-kb sequence of MiB300H01, *Glyma10g36600* was considered to be the strongest candidate for the *E2* locus based on the functional annotation in junction with the functional interpretations in previous genetic studies (Buzzel, 1971; Buzzel and Voldeng, 1980; Cober et al., 1996a).

The candidate *E2* gene was referred to as *GmGIa*. The coding sequence of *GmGIb*, the closest homolog of *GmGIa* in the genome, was also predicted.

The coding sequence of *GmGIa*-Mo from Moshidou Gong 503 containing 14 exons is prolonged to a 20-kb genomic region. Interestingly, the marker 2 derived from AFLP polymorphic band *E6*0M38 was located in the fifth intron and cosegregated with *E2* (Watanabe et al., 2011). Four SNPs were detected in the coding sequence of *GmGIa*-Mi, the Misuzudaizu early flowering allele, in comparison with *GmGIa*-Mo. Especially, an SNP in the 10th exon resulted in a premature stop codon mutation leading to a truncated 521 AA GI protein in *GmGIa*-Mi. Considering this stop codon mutation is functional in *GmGIa*, a derived amplified polymorphic sequence (dCAPs) marker was developed to genotype other corresponding NILs of Harosoy (*e2*/*e2*). The genotypes of the *E2* in all NILs tested were completely consistent with the genotypes of this dCAPs marker. This result further verified the candidacy of *GmGIa* for the *E2* loci and that this conserved stop codon mutation was a causal factor for the early flowering phenotype (Watanabe et al., 2011). To further validate whether mutations in the *GmGIa* can cause profound impact on flowering time and maturity, we identified a mutant line from X-ray-irradiated and ethyl methanesulfonate (EMS)-derived libraries by TILLING (McCallum et al., 2000). In comparison with wild-type cultivar Bay carrying the *E2* allele, the mutant line whose *E2* gene had a deletion in the 10th exon leading to a truncated protein (735 amino acids) showed a significant earlier (8 days) flowering phenotype under natural day-length conditions (Watanabe et al., 2011).

Taken together, *GmGIa* is the responsible gene for the *E2* locus. Refer to the formal publication on the positional cloning of the *E2* gene (Watanabe et al., 2011) for the detailed cloning procedure.

Three *GmGIa* haplotypes (H1, H2, and H3) were identified amid cultivated cultivars and their wild relatives in soybean. Interestingly, additional 44 haplotypes occur in wild soybeans (Wang et al., 2016). In cultivated as well as wild-type soybeans, H2 often occur in the southern part of China, while H3 was constrained to areas adjacent to the northeast region of China. H1, a domesticated haplotype, is the variant of H2, which was found to be profoundly distributed among cultivated soybeans. Intriguingly, the ortholog of H1 was present only at a low frequency in wild populations from Yellow River (Wang et al., 2016).

## The Route to Successful Identification of the *E1* Gene

**FIGURE 6:**
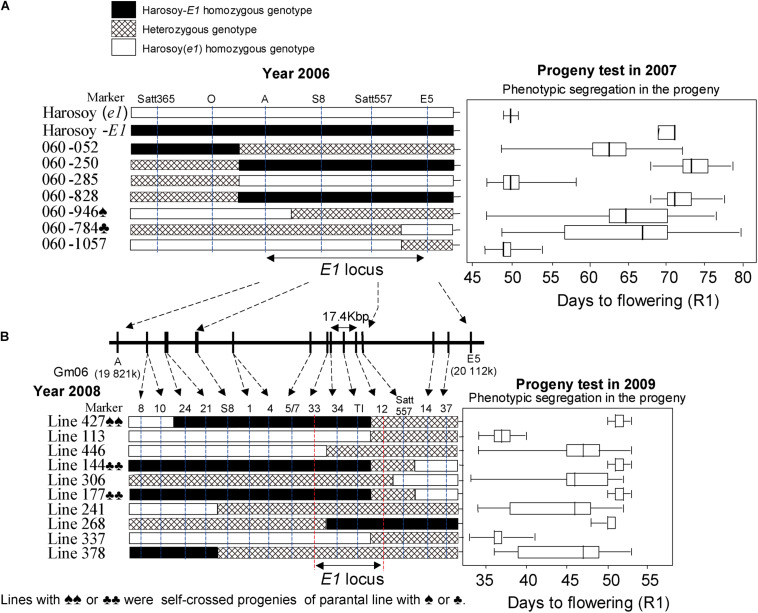
Fine mapping of the *E1* gene. Graphical genotypes of soybean recombinants carrying recombination in the *E1* region in the 2006–2009 experiments. Left: Recombinants detected. Right: Phenotypic segregation patterns in the progenies. Recombination is shown by white bars representing Harosoy (*e1*), black bars representing Harosoy-*E1*, and cross-hatched bars representing the heterozygote. The phenotypic segregation is presented in boxplot format. The box, the bold vertical line, and the horizontal line, respectively represent the interquartile region, median, and range of flowering time. In 2006–2007 **(A)**, with seven recombinants, we were able to delimit the *E1* to a 289-kb region. In 2008–2009 **(B)**, with 10 recombinants, we further delimited the *E1* region to a 17.4-kb region (adapted from Xia et al., 2012).

The RHL1-156 line with a heterozygous segment (approximately 17 cM) comprising the *E1* locus was screened out from the RILs population derived from a cross between Misuzudaizu and Moshidou Gong 503. Importantly, all other flowering-time-related QTL loci (except for the *E1* locus) anchoring segments were homozygous in this line. Upon segregation, a population of 1,006 individuals was derived from the RHL1-156. The *E1* locus could be mapped between Satt365 and GM169, at the distances of about 0.1 and 0.4 cM. The *E1* locus is located in the pericentromeric region of chromosome 6 in soybean^1^, with a high ratio of physical to genetic distance. Accordingly, no polymorphic AFLP bands had been detected between bulks of *E1* and *e1*, thus fine mapping halted due to the lack of molecular marker. It was difficult to develop new molecular markers in the era before the genome information publically available. Therefore, we shifted the cloning strategy and generated a mapping population of Harosoy-*E1* (*E1e2E3E4e5*) × Harosoy(*e1*) (*e1e2E3E4e5*), both of which carry identical genetic background except the *E1* locus. Flowering times of Harosoy-*E1*, F1 plant, and Harosoy (*e1*) were 45.0 ± 0.78 days (mean ± SD), 41.5 ± 1.16 days, and 34.9 ± 0.83 days, respectively, at Matsudo, Japan (35°78′N, 139°90′E), in 2005. The results indicated that the effects of the *E1* locus were about 10 days, and the *E1* allele is partially dominant over *e1*. For the F2 population (117 plants), *E1* was initially mapped between markers Satt365 and Satt289 by means of QTL analysis of flowering time at Matsudo in 2005, and the closest marker was Satt557. Among an F2:3 population of 1442 plants derived from 51 F2 plants that were heterozygous at Satt557, seven recombinants between markers Satt365 and Satt289 were identified (Figure 6).

The segregation patterns of flowering time among its progeny in 2007 at Tsukuba, Japan (36°03′N, 140°04′E) were used to accurately estimate the *E1* genotype for each recombinant. Despite a physical distance of 133 kb, we could not detect any recombination event occurring between the markers S8 and Satt557, which might be ascribed to a low recombination rate occurring in the pericentromeric region.

Therefore, the *E1* region was only located to an interval of ∼289 kb between markers A and marker E5 (Figure 6). According to the prediction using RiceGAAS (Sakata et al., 2002), more than 40 genes were annotated for this 289-kb region (Figure 6). Therefore, a new round of fine mapping became necessary to further delimit the region of *E1*.

With the aid of a simple seed genotyping developed in the lab, 13,761 F2:5 seeds having a heterozygous *E1* background and 10 recombinants carrying crossovers within the 289-kb region were successfully screened out. Similarly, the phenotypic segregation pattern of the progeny was evaluated at Tsukuba in 2009 to judge the *E1* genotype of each recombinant (Figure 6).

The *E1* gene was delimited to the region between markers 12 and 33, judging by the fact that the phenotypes cosegregated with markers 34 and TI among these recombinants (Figure 6). Molecular markers of *E1* region were used to screen in two independent BAC libraries of Misuzudaizu and Williams 82. In order to construct the BAC contigs, BACs were selected for shotgun sequencing based on the presence of molecular markers including BAC end sequencing-derived makers and the fingerprinting profiling of each BAC clone digested with *Hin*dIII (Xia et al., 2005, 2009).

Sequences yield from single BAC were assembled individually, and two physical contigs were successfully built for Misuzudaizu and Williams 82, respectively. The delimited *E1* region corresponds to 17,372 bp in Misuzudaizu (dominant *E1*) and 22,876 bp in Williams 82 (recessive *E1*). In the 17,372 bp from Misuzudaizu and Harosoy-*e1*, a single intron-free gene (AB552962, 525 bp, 174 aa) was consistently annotated by various software, such as GenScan (Burge and Karlin, 1997), and was designated as the *E1* gene. In recessive *e1* cultivars of Williams 82 and Harosoy (*e1*), a single missense point mutation was detected in the coding region of *E1*, resulting in a change from threonine to arginine at AA 15. This recessive allele was referred as to *e1*-as (AB552963). In Sakamotowase and its derived NILs, a 1-bp deletion in codon 17 at the *E1* locus resulted in a premature stop, designated as *e1-fs* (AB552971).

In some early flowering cultivars such as Fiskeby V, Yukihomare, Toyosuzu, Toyomusume, Hejian 1, and Heihe 2, there was approximately 130 kb deletion (including the entire *E1* gene) and was designated *e1-nl*. Both in the growth chamber and in the field, cultivars with the *e1-as* genotype generally flowered and matured intermediate between the *E1* and *e1-fs* genotypes, demonstrating that *e1-as* is a leaky allele and retains partial *E1* function. The function of *E1* in delaying flowering was confirmed by the EMS-derived *E1* mutants showing early flowering phenotype.

The *E1* gene encodes a protein that contains a putative bipartite nuclear localization signal (NLS) and a B3 domain, suggesting that this protein is a transcription factor. This mutation from *E1* to *e1-as* occurs in the first basic domain (amino acid motif KKRK) of the putative bipartite NLS, which might affect nuclear targeting. Through analysis of transformed *Arabidopsis* protoplasts and onion cells, the *E1* protein was mainly localized in the nucleus, whereas the *e1-as* was found in the nucleus as well as in the cytoplasm. *E1* expression was highly repressed under both short- and long-day conditions in cultivars carrying *e3e3*/*e4e4*.

The *E1* expression level was negatively correlated with the transcriptional abundance of *FT2a* and *FT5a*, two homologs of *Arabidopsis FT* that promote flowering (Kong et al., 2010) under the regulation of the *E3* and *E4* loci (Xia et al., 2012). Refer to the formal publication on the positional cloning of the *E1* gene (Xia et al., 2012) for the detailed cloning procedure.

The molecular identification of *E1* for the repression of flowering at the *E1* locus represents a significant step forward in photoperiodic flowering and thus has implications in breeding programs and cultivation practices. The expression level of functional *E1* gene was strongly associated with flowering time (Zhai et al., 2015).

The soybean genome has two *E1* homologs, *E1La* (*Glyma04g24640*, *Glyma.04G156400.1*) and *E1Lb* (*Glyma18g22670*, renamed as *Glyma.04G143300.1*).

Under long-day conditions, the expressions of all three genes of Harosoy peaked before dusk and after dawn the next day. The transition between light and dark phases and night–break experiments revealed that *E1* family genes were expressed solely during light periods (Xu et al., 2015). In the cultivar “Toyomusume,” which lacks the *E1* gene, silencing of *E1La* and *E1Lb* resulted in the upregulation of the expression of *FT2a* and *FT5a* and early flowering phenotype. Thus, *E1La* and *E1Lb* might have similar function to *E1* in flowering (Xu et al., 2015). *E1Lb* suppresses flowering under long-day conditions by blocking the expression of *FT2a* and *FT5a* in a fashion independent of *E1* (Zhu et al., 2019). Regulation of *E1* and *E1L* expression by light is dominated by *E3* and *E4*, and regulation of *FT2a* and *FT5a* expression is controlled by *E1* and *E1L* (Xia et al., 2012; Xu et al., 2015). This module may be a major regulator in photoperiodic flowering of soybean (Xia et al., 2012; Xu et al., 2015), which is different from CO/FT module in *Arabidopsis* (Samach et al., 2000) and rice (Kojima et al., 2002).

The *E1* homolog *Phvul.009G204600* (*PvE1L*) from common bean, a short-day leguminous species, was proven to delay the onset of flowering in soybean (Zhang et al., 2016). However, *Medtr2g058520*, the *E1* homolog from long-day leguminous species, promotes flowering (Zhang et al., 2016). Thus, the functional conservation and diversification of *E1* family genes from legumes may be associated with lineage specification (Zhang et al., 2016).

Although both *FT2a* and *FT5a* are under the control of *E1*, and collectively regulate flowering time, the function of *FT2a* is more prominent in SD. However, *FT5a* functions more prominently in LD, which affects adaptability of soybean to high latitude (Kong et al., 2014; Takeshima et al., 2016). The *ef* allele at *FT5a* is a rare haplotype, conferring an adaptive option at latitudes when early flowering is needed (Cai et al., 2020). *FT4* and *FT1a* were proven to be repressing flowering, which are antagonistic to *FT2a* and *FT5a*. Both genes are expressed at higher levels under LD compared SD, indicating that both are induced by *E1* (Zhai et al., 2014a; Liu et al., 2018).

Soybean genome has 12 *FT*-like genes, which scattered in six homologous pairs, *FT1a*/*b*, *FT2a*/*b*, *FT2c*/*d*, *FT3a*/*b*, *FT5a*/*b*, and *FT4*/*6* (Wu et al., 2017). Evolutionary trajectories of duplicated *FT* homologs and their functional roles in soybean domestication were reported (Wang et al., 2015; Wu et al., 2017). The *FT2c* allele having a transposon insertion is widely spread in soybean landraces but not in domesticated soybean, indicating that this allele spreads at the beginning of soybean domestication (Wu et al., 2017). *FT2a* was identified to be responsible for *E9* (Zhao et al., 2016). Studies on the expression levels of different alleles among NILs and photoperiodic-insensitive cultivars indicated that the SORE-1 (a Ty1/copia-like retrotransposon) insertion in *E9* diminished *FT2a* expression (Zhao et al., 2016).

## Allelic Combinations of the *E1* to *E4* Loci Primarily Determine Latitudinal Distribution

**FIGURE 7:**
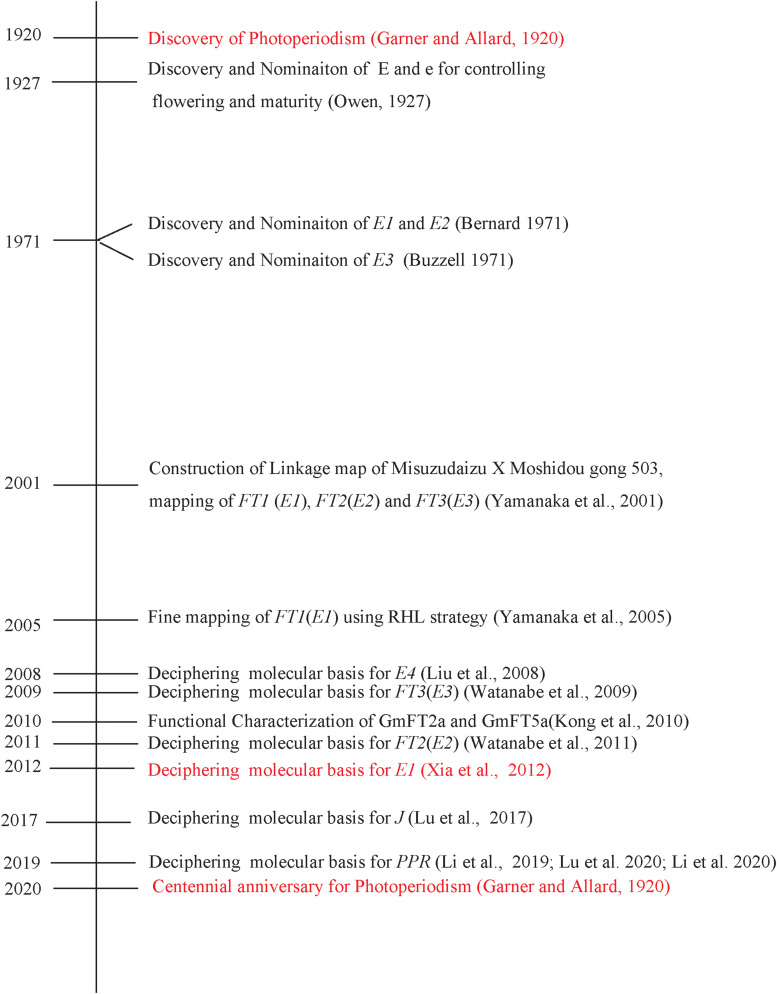
The important research events in soybean since the discovery of the photoperiodism.

*GmPhyA2*, another phytochrome A gene, was proven to be the causal gene for the *E4* locus by using a candidate gene approach (Liu et al., 2008). At the recessive allele (*E4-SORE-1*), the insertion of a Ty1/copia-like retrotransposon into exon 1 of the *E4* gene weakens the function of the *E4* gene on repressing flowering (Liu et al., 2008; Figure 7).

Natural variations in *E1*–*E4* genes were determined for 62 cultivars or landraces and a wild soybean accession (Tsubokura et al., 2014). Allelic combinations at the *E1*–*E4* loci are associated with ecological types, and 62–66% variation in flowering time could be explained by these loci (Tsubokura et al., 2014). The association of maturity group of soybean varieties and the adaptation to diverse ecological or latitudinal regions with allelic variation in *E1*–*E4* were also performed in China (Jiang et al., 2014; Zhai et al., 2014b), the United States (Langewisch et al., 2014, 2017; Wolfgang and Charles, 2017), and Europe (Kurasch et al., 2017).

Liu L. P. et al. (2020) reported that the allele combinations of *e1-as*/*e2-ns*/*e3-tr*/*E4*, *E1*/*e2-ns*/*E3*/*E4*, and *E1*/*E2*/*E3*/*E4* are dominant genotypes in the Northeast China, Huang-Huai-Hai (HHH) Rivers Valley, and South China regions, respectively. Notably, *E1* and *E2*, especially *E2*, affected flowering and variation maturity time of soybean significantly.

Among the soybean population at Novi Sad, Serbia, *e1-as*/*E2*/*E3*/*E4* was the most dominant genotype and presented the best performance in terms of yield. This allelic combination is putatively the optimal one suitable for the environments of Central-Eastern Europe (Miladinovic et al., 2018).

A total of 15 multilocus genotypes at the *E1*–*E4* loci were identified from 53 photoperiod-insensitive accessions. At either the *E3* or *E4* locus, a recessive allele was observed for all of the 53 accessions. A loss-of-function of *e1-fs* or *e1-nl* or hypomorphic *e1-as* allele at the *E1* locus always occurred when a dominant allele is present at the other loci (Xu et al., 2013).

Soybean RIL lines with various allele combinations at the *E1*, *E2*, *E3*, and *J* loci were field tested for days to flowering (DTF) and days to maturity (DTM) in short-day tropical environments in Ghana. The alleles of these genes interacted with each other for DTF but not for DTM. The mutant allele *J* and *E1* had profound impact on DTF and DTM (Miranda et al., 2020).

“Enrei” (*E1*/*e2*/*e3*/*E4*) is one of the leading cultivars in Japan. In order to expand the adaptability of “Enrei,” NILs for *E2* and *E3* were developed, and their flowering, maturity, seed productivity, and seed-quality traits were evaluated in five different locations (Yamada et al., 2012). The dominant alleles *E2* and *E3* were introduced from “Sachiyutaka” (*E1*/*E2*/*e3*/*E4*) and “Fukuyutaka” (*E1*/*E2*/*E3*/*E4*), respectively, by recurrent backcrosses based on the functional DNA markers. The modification of genotypes at maturity loci provides new varieties that are adaptive to environments of different latitudes while retaining almost the same seed quality as that of the original cultivar. Modification of maturity loci is underway for several other cultivars. *E1* and *E1La/b* were simultaneously silenced *via* RNA interference, and a super-early maturity line was developed that will adapt to high-latitude short-season regions (Liu L. et al., 2020). In addition, targeted mutations of soybean flowering genes by CRISPR/Cas9 technology to modify flowering and maturity have been reported for *FT2a* (Cai et al., 2018), for *FT2a* and *FT5a* (Cai et al., 2020), and for *E1* (Han et al., 2019).

## Identification of New Genes Controlling Flowering Time

A potential candidate gene for *E10* was proposed as *FT4* (Samanfar et al., 2017). *FT4*, a homolog of *FT*, is positively regulated by *E1* and was proven to function as a flowering repressor (Zhai et al., 2014a).

*E11* is a recently reported locus that influences both flowering time and maturity, and the most likely candidate is reported to be a soybean homolog of *LATE ELONGATED HYPOCOTYL* (*LHY*) (Wang et al., 2019). A homolog of *EARLYFLOWERING 3* (*GmELF3*) was identified as a gene for *J* locus (Lu et al., 2017; Yue et al., 2017). J protein physically associates with *E1* promoter and downregulates its transcription (Lu et al., 2017). The GmFLC-like protein can directly suppress the expression of *FT2a* by physically interacting with its promoter region. *GmFLC-like* might be involved in long-term low temperature-triggered late flowering by repressing *FT* gene expression. The result of treatments with various low temperature durations showed that *GmFLC-like* acts as a floral repressor (Lyu et al., 2020).

*GmAGL1* was proven to promote flowering possibly in a fashion of photoperiodic regulation. Overexpression of *GmAGL1* leads to early maturity, but no reduction occurs in seed traits or oil and protein contents (Zeng et al., 2018).

Analysis of variations in coding and non-coding regions of the *GmGBP1* genes in 278 soybean accessions showed that the shorter growth period might be largely ascribed to higher *GmGBP1* expression. In addition, RNA-interference-mediated downregulation of *GmGBP1* resulted in a longer growth period under different day lengths. It was showed that *GmGBP1* can act as a positive regulator of *FT2a* and *FT5a* to promote the expression of *GmFULc*, leading to early flowering under short-days (Zhao et al., 2018).

Two pairs of homologs *COL1a/b* and *COL2a/b* and other 22 *CO*-like genes have been identified in the soybean genome. Although the RNAi-mediated downregulation of *COL1a/b* could lead to the downregulation of *E1* (Wu et al., 2019), the function of *COL* genes in soybean has not been well understood. The mutant lacking *COL2b* putatively weakens the repression of flowering by cool temperature, in which the expressions of *E1*, *FT2a*, and *FT5a* have been altered (Zhang et al., 2020a, b).

Recently, a great progress has been made on connection of clock genes with *E1-FT*s, the major flowering pathway in soybean (Lu et al., 2017, 2020; Li Y. et al., 2019; Bu et al., 2021).

The QTLs, *qFT12-1*/*Gp12*/*Tof12* or *Gp11*/*Tof11*, in chromosomes 11 and 12 have been identified to be *GmPRR3a* and *GmPRR3b*, two homologs of *Arabidopsis PSEUDO-RESPONSE REGULATOR* (*PRR*) *3* (Li M. W. et al., 2019; Li Y. et al., 2019; Li et al., 2020; Lu et al., 2020). Through the *LHY* homologs, both *GmPRR3a* and *GmPRR3b* function to promote *E1* expression and thus delay flowering under long-days (Lu et al., 2020). The allelic variation in *GmPRR3b* has been widely chosen through modern breeding (Li Y. et al., 2019; Li et al., 2020). The causal SNP (Chr12:5520945) likely confers *GmPRR3b* a suitable level of activity, resulting in early flowering and vigorous growth. This functional variation is preferentially retained during breeding or improvement of landraces or cultivars. This gene, showing rhythmic and photoperiod-dependent expression, is specifically induced in LD and appears to act as a transcriptional repressor of *GmCCA1a*, which directly moderates *J/GmELF3a* to control flowering time (Li et al., 2020).

Overexpression of *GmPRR37* noticeably repressed the flowering of transgenic soybean in LD but not in SD (Wang et al., 2020). *GmPRR37* downregulated the expression of *FT2a* and *FT5a*, the flowering-promoting *FT* homologs, and upregulated *FT1a* expression, flowering-repressing *FT* homolog under long-day conditions (Wang et al., 2020).

The long-juvenile (LJ) trait can increase the vegetative phase under short-day conditions, ensuing higher yield and enabling expansion of cultivation in tropical regions. *J* locus, the major classical locus conferring the LJ trait, was identified as the ortholog of *A. thaliana EARLY FLOWERING 3* (*ELF3*), which depends genetically on the legume-specific flowering repressor *E1* (Lu et al., 2017; Yue et al., 2017). J protein physically associates with the *E1* promoter to downregulate its transcription, alleviating suppression of two important *FT* genes and promoting flowering under short-days (Lu et al., 2017).

Evening complex (EC) can be formed by both LUX1 and LUX2 by interacting with J, which promotes flowering redundantly. The EC represses the expression of *E1* and its homologs by binding to the LBS (a specific LUX binding site) of their promoters. Thus, *FT2a* and *FT5a* were abundantly produced to induce flowering in SD (Bu et al., 2021).

## Conclusion and Future Perspective

**FIGURE 8:**
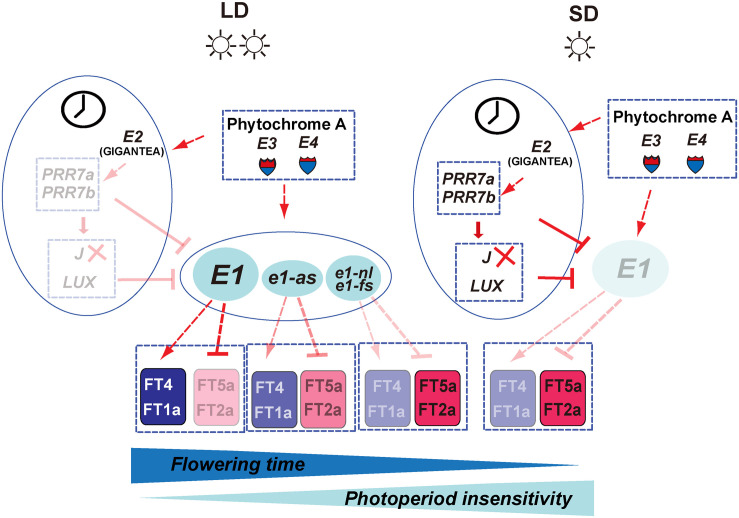
The putative flowering time gene network controlling the photoperiodic sensitivity in soybean. On the left panel, under long-day conditions, the expression of the *E1* gene is predominately promoted by the *E3* and *E4* genes. The elevated *E1* expression promotes the *FT4a* and *FT1a* expression and represses the *FT2a* and *FT5a*, leading to late flowering and higher photoperiod sensitivity. However, leaky allele *e1-as* displays partial function of the *E1* gene, and the non-functional allele, *e1-nl* or *e1-fs*, totally loses the promotion activity for the expression of *FT4a* and *FT1a* as well as the suppression activity for the expression of *FT2a* and *FT5a*. In addition, circadian clock genes such as *E2* as well as several downstream components such as *PRR3/7a*, *PRR3/7b*, LUX, and *J* are proven to participate in the control of *E1* expression. Under short-day condition, *E1* is strongly suppressed and leads to promoted expression of the *FT2a*/*FT5a* and early flowering time. The solid and dotted lines, respectively represent direct and indirect regulations. The arrow and T shape represent positive and negative regulation, respectively.

To mark the centennial of photoperiodism, we reviewed our efforts toward successful cloning of responsible genes at the major maturity loci *E1*, *E2*, and *E3*. Indeed, international efforts have been made including the discovery of the genetic factor controlling flowering and maturity, nomination, development of NIL, construction of linkage maps and BAC libraries, QTL mapping, fine mapping, and positional cloning using RHL and NIL. Since the successful identification of molecular basis of *E1*, *E2*, and *E3* genes, great progress has been made in identification of new genes that control or regulate flowering time and maturity and in flowering time gene networks especially related to circadian clock (Figures 7, 8). The central role of *E1* gene in photoperiodic flowering has been recently understood at molecular level. Both *E3* and *E4* genes mediate flowering responses under high ratio of R and FR light. Under LD, the *E3* and *E4* genes induce the expression of *E1* and *E1Lb*. *PRR3a* and *PRR3b* inhibit the expression of *GmLHY*/*GmCCA1* by binding to their promoters. Furthermore, *GmLHY* and *GmCCA1* can bind to the *E1* promoter and thus suppress its expression. *E1* can essentially repress the expression of flowering-inducing factors *FT2a* and *FT5a* and promote the expression of flowering-inhibitory factors *FT4* and *FT1a*. As a result, flowering is delayed under LD. Under SD, the functions of *E3* and *E4* are greatly weakened, leading to a suppressed expression of the *E1*. Meanwhile, *J* can inhibit *E1* expression. Consequently, the *E1* expression is strongly repressed in SD. The repressing effect of *FT2a* and *FT5a* by *E1* is strongly alleviated; in contrast, the expression of *FT1a* and *FT4* is suppressed (Figure 8). Therefore, flowering is strongly promoted in SD.

To date, the draft flowering time gene network of Phytochrome-clock-related gene *E1-FT*s has been built. However, the detailed regulatory mechanism remains poorly understood. Although the *E1* gene stands as a key hub gene in the regulation of flowering time in soybean, its pleiotropic function on other agronomic or phenotypic traits has not been well exploited. We also needed to clarify the functions of large numbers of flowering time gene homologs present in soybean genome, as well as their functional diversification and evolution in relation to domestication and modern breeding. Further identification of important components of *E1* pathway and studies on the detailed and coordinate regulation of flowering time gene network starting from the light reception to the full maturity will enable us to understand the nature of photoperiodism at molecular level in soybean.

## Author Contributions

ZX, SW, and KH: conceptualization and writing (review and editing). HZ, HW, and KX: investigation and visualization. All authors contributed to the article and approved the submitted version.

## Conflict of Interest

The authors declare that the research was conducted in the absence of any commercial or financial relationships that could be construed as a potential conflict of interest.

## Footnotes

1 http://www.phytozome.net
